# Variation among hospitals in the continuity of care for older hospitalized patients: a cross-sectional cohort study

**DOI:** 10.1186/s12913-021-06584-0

**Published:** 2021-06-05

**Authors:** James S. Goodwin, Shuang Li, Jie Zhou, Yong-Fang Kuo, Ann Nattinger

**Affiliations:** 1 Sealy Center on Aging, University of Texas Medical Branch, University Blvd, Galveston, TX 77555-0177 USA; 2 Medical College of Wisconsin, Milwaukee, WI USA

**Keywords:** Hospitalist, Hospital admission, Medicare, Continuity of care

## Abstract

**Background:**

Little is known about how continuity of care for hospitalized patients varies among hospitals. We describe the number of different general internal medicine physicians seeing hospitalized patients during a medical admission and how that varies by hospital.

**Methods:**

We conducted a retrospective study of a national 20% sample of Medicare inpatients from 01/01/16 to 12/31/18. In patients with routine medical admissions (length of stay of 3–6 days, no Intensive Care Unit stay, and seen by only one generalist per day), we assessed odds of receiving all generalist care from one generalist. We calculated rates for each hospital, adjusting for patient and hospital characteristics in a multi-level logistic regression model.

**Results:**

Among routine medical admissions with 3- to 6-day stays, only 43.1% received all their generalist care from the same physician. In those with a 3-day stay, 50.1% had one generalist providing care vs. 30.8% in those with a 6-day stay. In a two-level (admission and hospital) logistic regression model controlling for patient characteristics and length of stay, the odds of seeing just one generalist did not vary greatly by patient characteristics such as age, race/ethnicity, comorbidity or reason for admission. There were large variations in continuity of care among different hospitals and geographic areas. In the highest decile of hospitals, the adjusted mean percentage of patients receiving all generalist care from one physician was > 84.1%, vs. < 24.1% in the lowest decile. This large degree of variation persisted when hospitals were stratified by size, ownership, location or teaching status.

**Conclusions:**

Continuity of care provided by generalist physicians to medical inpatients varies widely among hospitals. The impact of this variation on quality of care is unknown.

**Supplementary Information:**

The online version contains supplementary material available at 10.1186/s12913-021-06584-0.

## Introduction

Provider continuity is an important aspect of medical care, associated with better health, fewer hospitalizations, higher quality of life and other important health outcomes [1–10]. Continuity of care can be important both in outpatient care and across community to hospital transitions. By 2006, most hospitalized Medicare patients did not receive any care from a physician who had previously provided care for them [7], and such discontinuity is associated with worse outcomes [8, 9].

Continuity of the care provided to hospitalized patients has been less well studied [10]. Recent studies have suggested that patients admitted for medical illness are likely to be cared for by more than one general internist during their stay [11]. Such discontinuity seems driven by the growth of care by hospitalists, and the fact that many hospitalists have work schedules that do not allow for continuity of care [11]. Just as with outpatient medicine, hospitalized patients who experience discontinuities in their care also have worse outcomes [12].

In this report, we analyze continuity of care in older patients hospitalized for a medical diagnosis, using a 20% sample of national Medicare data from 2016 to 2018. We calculate the number of different generalist physicians providing care for a patient, and how that varies by length of stay and patient characteristics. We also present the variation among hospitals and among hospital referral regions (HRRs) in the average number of different generalist physicians providing care, adjusted for length of stay, admitting diagnosis and other patient characteristics. We hypothesized that inpatient continuity would decline over time, that it would be only weakly related to patient characteristics such as age, sex, and race/ethnicity, but vary widely among hospitals and by type of hospital.

## Methods

### Data sources

We used 20% national Medicare claims for January 1, 2016 through December 31, 2018 in the analyses, including the Medicare Denominator File, the Carrier File, the Outpatient Statistical Analysis File, and the Medicare Provider Analysis and Review File.

### Study population

The cohorts are presented in Supplementary eFig. 1. We first identified all hospital admissions discharged alive from January 1, 2016 to December 31, 2018 who received care from at least one generalist physician (general internal medicine, family medicine, geriatrics, hospitalist). To reduce heterogeneity, we restricted the cohort to those with a 3-, 4-, 5- or 6-day length of stay. To allow for a year of data prior to admission to assess comorbidity, we included only hospitalizations for enrollees aged 66+ years with a medical diagnostic-related group (DRG) code. We also eliminated admissions with an Intensive Care Unit (ICU) stay and excluded those hospitalizations that were billed for generalist physician services more than once during any day, because both are associated with greater illness severity or the occurrence of a complication in the hospital. This might lead to care from different generalist physicians (e.g., an on-call hospitalist at night), but not reflect the underlying care patterns experienced by “routine medical admissions.” To ensure complete information on comorbidity, we restricted the sample to those with Parts A and B Medicare without a health maintenance organization enrollment in the 12 months prior to the hospitalization, in order to be able to capture diagnoses in the prior year.

### Admission characteristics

The Medicare Denominator File was used to extract information on patient age, sex and race/ethnicity (non-Hispanic white, non-Hispanic black, Hispanic, other). Medicaid eligibility was measured using the state buy-in information in the Medicare Denominator File. The percentage of high school graduates in the patients’ ZIP Code area was obtained from the 2017 American Community Survey estimates of the US Census Bureau. Elixhauser comorbidities were assessed based on outpatient, inpatient and carrier claims in the 12 months prior to the hospital admission [13]. Medicare Provider Analysis and Review claims were used to determine the length of stay, DRG, ICU utilization, residence prior to hospitalization (community vs. nursing facility/institution), emergency hospitalization, weekend hospitalization and the number of hospitalizations in the prior 12 months.

### Hospital characteristics

Information on hospital bed size, location (urban/rural), type (for profit, nonprofit, public) and medical school affiliation (major, limited, graduate, no affiliation) was extracted from the Provider of Service files [14]. The percentage of Medicaid admissions was calculated from cohort selection step 3 in Supplementary eFigure 1.

### Analyses

We described the number of different generalist physicians submitting evaluation and management charges for a patient during an admission, stratified by length of stay. We then constructed multilevel logistic regression models to generate odds of receiving care from just one generalist physician, adjusted for patient characteristics and length of stay. One model included admission and hospital levels. With this model, we assessed the association of hospital characteristics with the odds of receiving continuity of care. Another model included admission and HRR. We calculated intraclass correlation coefficients (ICCs) from the null models and in models including admission and hospital characteristics, and graphed the adjusted percentage for each hospital or for each HRR of medical admissions receiving care from only one generalist, adjusted for admission characteristics and length of stay. All analyses were performed using SAS version 9.4 (SAS Institute, Cary, NC).

## Results

Table 1 presents the number of medical admissions among Medicare enrollees age 66 or older with a 3-, 4-, 5- or 6-day length of stay stratified by how many different generalists submitted charges for their inpatient care. This was limited to admissions with no ICU stay and where there was a maximum of one generalist charge per day. Overall, 43.1% of admissions received care from only one generalist, with 15.7% receiving care from three or more generalists during their stay. The percentage of admissions seeing only one generalist declined substantially with increasing length of stay, from 50.1% for a 3-day stay to 30.8% for a 6-day stay.

**Table 1:** Number and percentage of Medical admissions receiving care from 1, 2, or 3+ generalist physicians. Results are stratified by different lengths of stay, from a 20% sample of US fee-for-service Medicare enrollees from 1/1/16 to 12/31/18. The cohort is limited to admissions with a medical Diagnostic Related Group admission, with no Intensive Care Unit stay and excluding those receiving any care from generalist physicians twice in one day. Generalist physicians include general internal medicine, family medicine or geriatrics

Length of stay	Number of hospitalizations	Number of generalists		
		*N* = 1	*N* = 2	*N* = 3+
**3 days**	315,555	158,166 (50.1%)	127,372 (40.4%)	30,017 (9.5%)
**4 days**	202,754	85,037 (41.9%)	85,105 (42.0%)	32,612 (16.1%)
**5 days**	127,899	45,416 (35.5%)	53,717 (42.0%)	28,766 (22.5%)
**6 days**	83,209	25,596 (30.8%)	34,322 (41.2%)	23,291 (28.0%)
**Total**	729,417	314,215 (43.1%)	300,516 (41.2%)	114,686 (15.7%)

Table 2 presents the unadjusted rates of seeing just one generalist during a routine medical admission, by admission characteristics. Also shown are the odds of seeing just one generalist, adjusted for other admission characteristics and also for clustering of admissions in hospitals, in a multilevel multiple logistic regression model (Model 1 in Table 2). In both unadjusted and adjusted analyses, there was a strong relationship with length of stay, with 6-day admissions only 38% as likely as 3-day admissions to receive care from just one generalist (Odds Ratio [OR = 0.38, 95% Confidence Interval [CI] = 0.38, 0.39). Admissions from nursing facilities were less likely to see just one generalist (OR = 0.94 CI, 95% CI = 0.92, 0.96), as were admissions from the emergency room (OR = 0.77; 0.75, 0.78) or those on weekends (OR = 0.73; 0.73, 0.74). There was very little influence of other patient characteristics, with the odds of seeing only one generalist varying only minimally by age, gender, race/ethnicity, education, Medicaid eligibility or number of prior hospitalizations. We also included the DRG-Major Diagnostic Category and individual patient comorbidities in this model, and they are shown in Supplementary eTable 2. The differences in odds of receiving care from one generalist by any pre-existing comorbidity were not large. There was some variation by DRG-Major Diagnostic Category, with admissions for mental disorders and burns more likely to be cared for by just one generalist. Odds of seeing just one generalist declined from 2016 to 2018.

**Table 2:** Odds of receiving care from only one generalist physician. Results are for admissions aged 66+ without an Intensive Care Unit stay during hospitalization, adjusted for patient characteristics, from a two level logistic regression model (hospital and admission), among a 20% sample of US fee-for-service Medicare enrollees age > 66 years, from 1/1/16 to 12/31/18

Characteristic	N (%)	Observed rate	Model 1 Odds Ratio^a^ (95% Confidence Interval)	Model 2 Odds Ratio^a^ (95% Confidence Interval)
All	729,417	43.1%		
Age (Per year)				
Q1 (> = 66; <=73)	188,231 (25.8%)	42.8%	Reference	Reference
Q2 (> = 74; <=80)	182,726 (25.1%)	43.1%	1.00 (0.99–1.02)	1.00 (0.99–1.02)
Q3 (> = 81; <=87)	189,102 (25.9%)	43.1%	1.01 (0.99–1.03)	1.01 (0.99–1.03)
Q4 (> = 87)	169,358 (23.2%)	43.3%	1.03 (1.01–1.05)	1.03 (1.01–1.04)
Education (Percent of persons age 25+ in Zip area with high school education) (per percent)				
Q1 (<=82.8)	183,360 (25.1%)	48.2%	Reference	Reference
Q2 (> = 82.9; <=88.7)	182,269 (25.0%)	44.0%	0.99 (0.98–1.01)	0.99 (0.98–1.01)
Q3 (> = 88.8; <=93.0)	183,500 (25.2%)	41.1%	0.97 (0.96–0.99)	0.98 (0.96–0.99)
Q4 (> = 93.1)	180,288 (24.7%)	38.9%	0.98 (0.96–0.99)	0.98 (0.97–1.01)
Year				
2016	239,223 (32.8%)	44.4%	Reference	Reference
2017	247,960 (34.0%)	43.1%	0.96 (0.95–0.97)	0.96 (0.95–0.97)
2018	242,234 (33.2%)	41.7%	0.91 (0.90–0.92)	0.91 (0.90–0.92)
Gender				
Female	440,343 (60.4%)	43.5%	Reference	Reference
Male	289,074 (39.6%)	42.5%	1.01 (1.01–1.02)	1.01 (1.01–1.02)
Medicaid				
No	570,166 (78.2%)	42.3%	Reference	Reference
Yes	159,251 (21.8%)	46.0%	1.01 (0.99–1.03)	1.01 (0.99–1.03)
Race				
White	612,701 (84.0%)	42.3%	Reference	Reference
Black	64,030 (8.8%)	46.4%	1.03 (1.01–1.05)	1.03 (1.01–1.05)
Hispanic	30,267 (4.1%)	49.9%	0.99 (0.96–1.02)	0.99 (0.97–1.02)
Other	22,419 (3.1%)	45.0%	1.02 (0.98–1.05)	1.02 (0.99–1.05)
Residence prior to hospitalization				
Community	667,721 (91.5%)	43.2%	Reference	Reference
Nursing facility or other institution	61,696 (8.5%)	42.1%	0.94 (0.92–0.96)	0.94 (0.92–0.96)
Length of stay				
3 days	315,555 (43.2%)	50.1%	Reference	Reference
4 days	202,754 (27.8%)	41.9%	0.68 (0.67–0.69)	0.68 (0.67–0.69)
5 days	127,899 (17.5%)	35.5%	0.49 (0.48–0.50)	0.50 (0.49–0.50)
6 days	83,209 (11.4%)	30.8%	0.38 (0.38–0.39)	0.39 (0.38–0.39)
Number of hospitalizations in 12 months before admission (per hospitalization)				
0	335,465 (46.0%)	42.8%	Reference	Reference
1	186,403 (25.6%)	42.8%	1.01 (0.99–1.02)	1.01 (0.99–1.02)
2 and 3	147,014 (20.2%)	43.5%	1.03 (1.02–1.05)	1.03 (1.02–1.05)
4 and above	60,535 (8.3%)	44.3%	1.05 (1.02–1.07)	1.05 (1.02–1.07)
Emergency hospitalization				
No	143,678 (19.7%)	50.0%	Reference	Reference
Yes	585,739 (80.3%)	41.4%	0.77 (0.75–0.78)	0.77 (0.76–0.79)
Weekend hospitalization				
No	505,956 (69.4%)	45.2%	Reference	Reference
Yes	223,461 (30.6%)	38.3%	0.73 (0.73–0.74)	0.73 (0.73–0.74)
Bed size				
> 500	169,656 (23.3%)	39.9%	–	Reference
201–500	314,367 (43.1%)	40.5%	–	1.04 (0.91–1.20)
<=200	245,394 (33.6%)	48.6%	–	1.37 (1.19–1.58)
Location				
Rural	138,481 (19.0%)	53.1%	–	Reference
Urban	590,936 (81.0%)	40.7%	–	0.64 (0.59–0.70)
Type of provider				
For profit	96,302 (13.3%)	52.1%	–	Reference
Public	97,306 (13.3%)	48.3%	–	0.82 (0.74–0.92)
Non-profit	535,809 (73.4%)	40.5%	–	0.54 (0.49, 0.59)
Medical school affiliation				
Major	143,993 (19.7%)	41.3%	–	Reference
Limited	144,656 (19.8%)	40.0%	–	0.80 (0.69–0.92)
Graduate	31,784 (4.4%)	38.1%	–	0.83 (0.66, 1.04)
No affiliation	408,984 (56.1%)	45.2%	–	0.96 (0.85, 1.10)

^a^Odds ratio are from multilevel model (admission and hospital) adjusted for all characteristics presented in the table, as well as the 31 Elixhauser comorbidities (each entered separately), and the Diagnosis Related Group-Major Diagnostic Category (DRG-MDC) codes. Results for the comorbidities and DRG-MDC are presented in Supplementary eTable 1

Model 2 in Table 2 includes specific hospital characteristics in addition to admission characteristics. Hospital characteristics associated with increased odds of continuity of care include smaller size, rural location, for-profit status and major teaching hospital. Including hospital characteristics had minimal effect on the relative lack of association of admission characteristics with the odds of receiving care from just one generalist.

We estimated the variation among hospitals in continuity of care with ICCs. The ICC for the two-level null model was 0.29, indicating that 29% of the variation in whether a hospitalized patient experiences continuity of care is explained by which hospital they are in. It was unchanged when the admission characteristics listed in Table 2 and Supplementary eTable 1 were added (that is, Model 1 of Table 2). When the hospital characteristics listed in Table 2 were added, the ICC decreased to 0.26, suggesting that about 10% of the variation among hospitals in continuity of care is explained by those hospital characteristics.

**Fig. 1:**
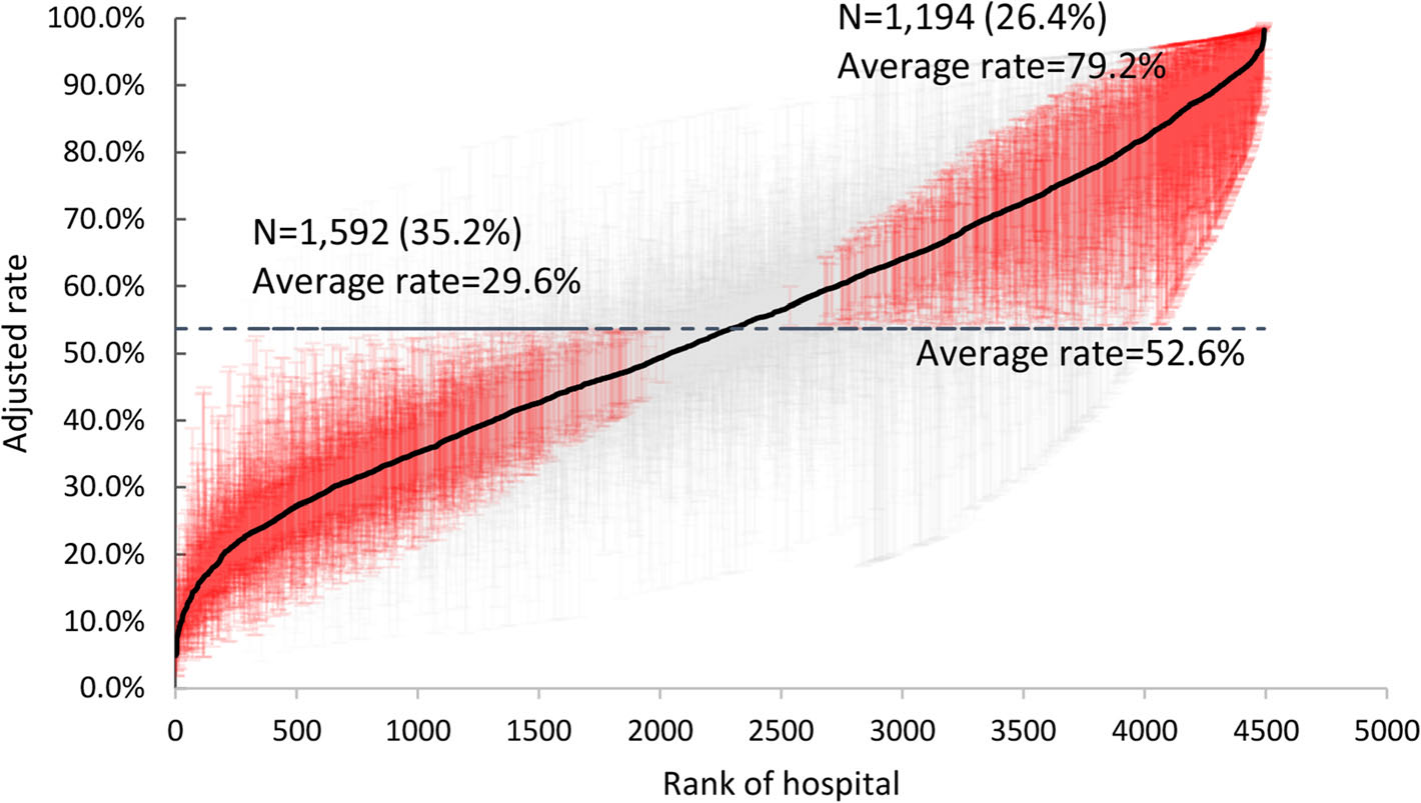
Adjusted rate of seeing one generalist during hospitalization, by hospital. The percent of patients in a hospital who see only one generalist during hospitalization, for 4523 US hospitals, from a multilevel logistic regression model (admission, hospital) adjusted for the patient characteristics listed in Table 2, plus Diagnosis Related Group-Major Diagnostic Categories (DRG-MDC) and the 31 Elixhauser comorbidities listed in Supplementary eTable 1. Hospitals with adjusted rates significantly different from the mean adjusted rate of all hospitals are shown in red, with the 95% confidence intervals

Figure 1 shows the distribution among 4523 US hospitals of the percent of patients who received care from just one generalist. The rates were adjusted for all the admission characteristics shown in Table 2 and Supplementary eTable 1. The 4523 hospitals are ordered from the hospital with the lowest rate of continuity, 3.6% of their patients, to the highest, 97.9%. There was substantial variation in continuity among the hospitals. The bottom 10% of hospitals all had rates less than 24.1%, and the top 10% of hospitals had rates greater than 84.1%. The hospitals with adjusted rates that are significantly higher or lower from the mean rate of all the hospitals are indicated in red; 26.4% of hospitals had significantly greater continuity with a mean of 79.2% of admissions seeing only one generalist, and 35.2% of hospitals had significantly lower continuity, with 29.6% of admissions seeing only one generalist.

**Fig. 2:**
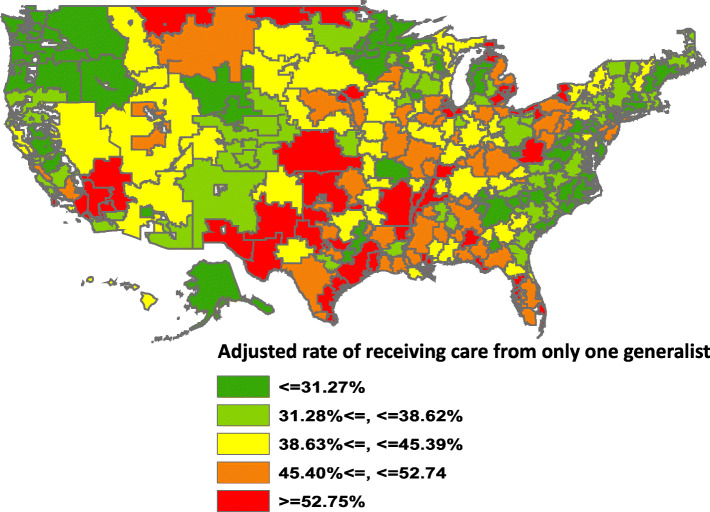
Adjusted rate of seeing one generalist during hospitalization, by Hospital Referral Region (HRR). Adjusted rate of seeing only one generalist during hospitalization, a multilevel logistic regression model (admission, HRR) adjusted for the patient characteristics listed in Table 2, plus Diagnosis Related Group-Major Diagnostic Categories and the 31 Elixhauser comorbidities listed in Supplementary eTable 1. The HRRs are color coded by the quintile of their adjusted mean rate of admissions seeing just one generalist physician. The intraclass correlation coefficient for the model used to generate the HRR adjusted rates was 0.071

There were also large geographic variations in continuity of care (Fig. 2). The mean adjusted rate of seeing only one hospitalist was < 32% in the lowest 20% of HRRs and was > 52% in the highest 20%. Rates appeared generally lower in New England, the mid-Atlantic states and the Northwest.

Table 3 presents the distributions of adjusted percent of admissions seeing only one generalist among 4523 US hospitals in the US, stratified by hospital characteristics. In the bottom 10% of all hospitals, the adjusted percent of routine medical admissions seeing only one generalist was < 24.1%, vs. > 84.1% for the top 10% of hospitals. When the hospitals were stratified by size, location, profit status or medical school affiliation, the breadth of the distributions was not largely affected (i.e., the distance between the 10th and 90th percentiles). However, larger hospitals and those with medical school affiliation had somewhat less variation than the comparison categories (Table 3). Supplementary eTable 2 presents the same analyses but limited to the 442 major teaching hospitals. There was still a large degree of variation in inpatient continuity of care when the analysis was limited to large major teaching hospitals.

**Table 3:** Distribution of 4523 US hospitals, stratified by hospital characteristics. Results show the adjusted percent of routine medical admissions who received all of their general medical care from one physician

	N hospital (%)	N admission (%)	Adjusted percent receiving care by one generalist^**a**^					
Hospital type			Mean	10th	25th	50th	75th	90th
**All hospitals**	4523	729,417 (100%)	52.6	24.1	34.9	51.5	69.9	84.1
**Bed size**								
> 500	367	169,656 (23.3%)	42.3	21.5	28.3	42.3	54.1	64.5
201–500	1091	314,367 (43.1%)	44.7	21.6	29.7	43.0	57.7	71.2
<=200	3065	245,394 (33.6%)	57.7	26.9	39.4	57.4	77.0	88.9
**Location**								
Rural	1870	138,481 (19.0%)	61.7	30.3	44.3	63.1	80.4	91.1
Urban	2653	590,936 (81.0%)	47.0	22.1	30.5	45.3	61.0	76.3
**Type of provider**								
For profit	760	96,302 (13.3%)	59.8	29.4	44.0	59.1	77.6	88.5
Public	1045	97,306 (13.3%)	61.3	28.4	43.6	63.2	80.8	90.9
Non-profit	2718	535,809 (73.4%)	49.4	23.6	33.1	48.0	64.5	78.7
**Medical school affiliation**								
Major	442	143,993 (19.7%)	45.8	23.3	31.3	45.5	58.4	69.2
Limited	627	144,656 (19.8%)	45.7	22.2	29.7	42.7	59.6	75.5
Graduate	126	31,784 (4.4%)	44.2	22.0	29.8	43.4	55.8	68.4
No affiliation	3328	408,984 (56.1%)	55.3	25.0	37.0	54.6	73.9	87.1

^a^Adjusted for admission characteristics in a two-level logistic regression model. Admission characteristics are listed in Table 2 and Supplementary eTable 1

## Discussion

Continuity of care has been threatened by an array of forces for at least two decades. These include the decrease in primary care physicians, changes in health insurance, the growth of specialization and the need to ensure easy and timely access to care [15–18]. Considerable attention has been paid to the discontinuities of care across the community to hospital and back to the community [1, 2, 7, 9].

Few studies have examined inpatient continuity of care in the hospital. Epstein et al. [19] studied a large national hospitalist group and reported that approximately 60% of admissions for pneumonia and heart failure saw at least two hospitalists. In a study of Medicare admissions for chronic obstructive pulmonary disease, congestive heart failure and pneumonia, we found that inpatient continuity of care provided by generalist physicians, including hospitalists, non-hospitalist generalists and primary care physicians, declined substantially between 1996 and 2006 [8].

Continuity of care for hospitalized patients is important for several reasons. It is unlikely that all relevant information communicated by patients and their families to a physician is included in the electronic medical record or is transmitted orally during hand-offs. In addition, information relevant to patient values and preferences and degree of family involvement can be key in medical and discharge decision making. Patients and their families may be less comfortable soliciting and following the advice of a physician they are seeing for the first time, particularly if the issue is value-laden such as end of life issues or discharge destination [4, 6, 20].

Few studies have explored associations of inpatient continuity of care with outcomes. A Joint Commission report blamed miscommunication between physicians during hospital hand-offs for the majority of serious adverse effects [21, 22]. Epstein et al. [19] reported that hospitalist discontinuity was associated with increased length of stay. In a single hospital study, hospitalist discontinuity was associated with a small increase in costs [23]. Another study of 474 admissions to a single hospital found no relationship of discontinuity with adverse events [24]. It is difficult to interpret the results of such studies because of potential biases. For example, hospital complications might lead to discontinuity because of care from an on-call physician and might also lead to increased length of stay and worse outcomes. In a recent study [12], we attempted to control for that bias by assessing the association of the working schedules of hospital physicians with the outcomes of patients under their care. We found that admissions cared for by hospitalists who usually worked several days in a row experienced lower post-discharge mortality, readmissions and costs than did admissions cared for by hospitalists with more intermittent schedules [12].

In the current study, we included medical admissions receiving care from any generalist physician (general internal medicine, family medicine, geriatrics), whether or not they were hospitalists. The diagnoses were predominately respiratory, cardiovascular, renal and gastroenterology (Supplementary eTable 1). We limited the sample to what we termed routine medical admissions, eliminating those with an ICU stay or who had received care from two generalist physicians on the same day, in order to better describe the usual care patterns of patients hospitalized for medical conditions. The variation among hospitals in continuity of care was substantial; in a two-level model, 29% of the variation in whether admissions were cared for by just one generalist was attributed to which hospital they were in. Conversely, the characteristics of the admission contributed very little to the variation in continuity. Adding admission characteristics did not measurably change the ICC. The 4523 hospitals in the analyses were highly heterogeneous in size, location, ownership and academic affiliation. However, these characteristics explained only about 10% of the variation among hospitals, and this variation was substantial even after stratifying by hospital type (Table 3).

Limitations of the analyses include their reliance on Medicare fee-for-service data, which excludes older individuals enrolled in Medicare health maintenance organizations. In addition, it is possible that differences in patients among hospitals contributed to the variation in continuity. However, adjusting for age, diagnoses, comorbidity and other factors had very little effect on estimates of continuity.

## Conclusions

In conclusion, there is great variability in the US in the likelihood that hospitalized Medicare patients will receive all their hospital care from one physician, and much of the variation is driven by the hospital to which the patient is admitted. The large degree of variation even among similar types of hospitals, such as major teaching hospitals, suggests that much of the variation in continuity of care is discretionary. Additional studies should examine the impact of these differences in continuity, if any, on quality of care and outcomes.

## Supplementary Information

**Additional file 1: Supplementary eFigure 1.** Cohort selection. **Supplementary eTable 1.** Other factors included in the analysis presented in Table 2. The list includes the Diagnosis Related Groups Major Diagnostic Categories (DRG-MDC) of the patient and the presence of any 31 comorbidities in the prior year. **Supplementary eTable 2.** The adjusted percent of routine medical admissions receiving all general medical care from one physician. The analysis is similar to Table 3, except the sample is limited to the 442 major teaching hospitals.

## Data Availability

The Medicare datasets used in this study are in the Virtual Research Data Center (VRDC) of the Center for Medicare and Medicaid Services (CMS). The authors analyzed all data within the CMS site and, as part of the Data Use Agreement (DUA), are not allowed to download VRDC data. The datasets used and/or analyzed during the current study are available from CMS after approval of a DUA and payment of a user fee to CMS.

## Abbreviations

HRR: Hospital Referral Region
DRG: Diagnosis Related Group
ICU: Intensive Care Unit
CI: Confidence Interval
OR: odds ratio
ICC: intraclass correlation coefficient
VRDC: Virtual Research Data Center
CMS: Center for Medicare and Medicaid Services
