# Supplementary material for: Variation among hospitals in the continuity of care for older hospitalized patients: a cross-sectional cohort study
Source: BMC Health Serv Res. 2021 Jun 5;21:552. doi: 10.1186/s12913-021-06584-0 (PMC8180074; doi:10.1186/s12913-021-06584-0)
Supplement: Supplementary file 1 — Additional file 1: Supplementary eFigure 1. Cohort selection. Supplementary eTable 1. Other factors included in the analysis presented in Table 2. The list includes the Diagnosis Related Groups Major Diagnostic Categories (DRG-MDC) of the patient and the presence of any 31 comorbidities in the prior year. Supplementary eTable 2. The adjusted percent of routine medical admissions receiving all general medical care from one physician. The analysis is similar to Table 3, except the sample is limited to the 442 major teaching hospitals. [file 12913_2021_6584_MOESM1_ESM.docx]

**Supplementary Online-Only Material.**

**Supplementary eFigure 1. Cohort selection.**

**Supplementary eTable 1. Other factors included in the analysis presented in Table 2.** The list includes the Diagnosis Related Groups Major Diagnostic Categories (DRG-MDC) of the patient and the presence of any 31 comorbidities in the prior year.

**Supplementary eTable 2. The adjusted percent of routine medical admissions receiving all general medical care from one physician.** The analysis is similar to Table 3, except the sample is limited to the 442 major teaching hospitals.

**Supplementary eFigure 1. Cohort selection.**

Step 6. All admissions who receiving any generalist care N=1,032,934 (81.73%)

Step 1. All acute hospitalizations from 01/01/2016 to 12/30/2018, length of stay between 3 and 6 days, inclusive N=3,951,625 (100%)

Step 2. Keep patients with complete Part A&B, no Health Maintenance Organization in the 12 months prior N=2,487,352 (62.95%)

Step 3. Keep patients aged 66+ as of hospitalization admission N=2,031,828 (81.69%)

Step 4. Keep hospitalization with non-Intensive Care Unit stay N=1,277,378 (62.87%)

Step 5. Exclude discharge death N=1,263,840 (98.94%)

Step 7. Keep Medical Diagnostic Related Group hospitalization N=814,036 (78.81%)

Step 9. Keep the hospitalizations with complete data N=729,417 (98.39%)

Step 8. Exclude admission with more than 1 generalist evaluation and management claims on any day N=741,344 (91.07%)

**Supplementary eTable 1.** **Other factors included in the analysis presented in Table 2.** The list includes the Diagnosis Related Groups Major Diagnostic Categories (DRG-MDC) of the patient and the presence of any 31 comorbidities in the prior year.

| Characteristic | N (%) | Observed rate | Odds Ratio^*^ (95% Confidence Interval) |
| --- | --- | --- | --- |
| All | 729,417 | 43.1% |  |

| DRG-MDC |  |  |  |
| --- | --- | --- | --- |
| Diseases and Disorders of the Nervous System | 51,003 (7.0%) | 42.5% | Reference |
| Diseases and Disorders of the Eye | 756 (0.1%) | 42.6% | 0.88 (0.75-1.03) |
| Diseases and Disorders of the Ear, Nose, Mouth and Throat | 5707 (0.8%) | 47.0% | 1.07 (1.01-1.14) |
| Diseases and Disorders of the Respiratory System | 142,860 (19.6%) | 44.4% | 0.98 (0.96-1.01) |
| Diseases and Disorders of the Circulatory System | 104,902 (14.4%) | 43.6% | 1.01 (0.99-1.04) |
| Diseases and Disorders of the Digestive System | 85,692 (11.8%) | 42.1% | 0.94 (0.92-0.97) |
| Diseases and Disorders of the Hepatobiliary System and Pancreas | 18,546 (2.5%) | 39.3% | 0.89 (0.86-0.92) |
| Diseases and Disorders of the Musculoskeletal System and Connective Tissue | 50,579 (6.9%) | 45.4% | 1.03 (0.99-1.06) |
| Diseases and Disorders of the Skin, Subcutaneous Tissue and Breast | 30,372 (4.2%) | 45.2% | 0.99 (0.96-1.02) |
| Diseases and Disorders of the Endocrine, Nutritional and Metabolic System | 28,710 (3.9%) | 47.0% | 1.04 (1.01-1.08) |
| Diseases and Disorders of the Kidney and Urinary Tract | 95,186 (13.1%) | 43.3% | 0.96 (0.93-0.98) |
| Diseases and Disorders of the Male Reproductive System | 1,792 (0.3%) | 43.1% | 0.93 (0.84-1.04) |
| Diseases and Disorders of the Female Reproductive System | 965 (0.1%) | 43.8% | 0.97 (0.84-1.11) |
| Diseases and Disorders of the Blood and Blood Forming Organs and Immunological Disorders | 12,450 (1.7%) | 45.0% | 1.02 (0.98-1.07) |
| Myeloproliferative Diseases and Disorders (Poorly Differentiated Neoplasms) | 3,871 (0.5%) | 48.6% | 1.26 (1.17-1.36) |
| Infectious and Parasitic Diseases and Disorders (Systemic or unspecified sites) | 74,965 (10.3%) | 35.8% | 0.83 (0.81-0.85) |
| Mental Diseases and Disorders | 5,586 (0.8%) | 51.0% | 1.29 (1.21-1.38) |
| Alcohol/Drug use or induced mental disorders | 2,077 (0.3%) | 49.7% | 1.33 (1.20-1.47) |
| Injuries, Poison and Toxic Effect of Drugs | 5,518 (0.7%) | 43.6% | 1.03 (0.96-1.09) |
| Burns | 119 (0.0%) | 56.3% | 1.59 (1.06-2.38) |
| Factors Influencing Health Status and Other Contacts with Health Services | 7,326 (1.0%) | 50.9% | 1.12 (1.06-1.18) |
| Multiple Significant Trauma | 645 (0.1%) | 40.6% | 0.94 (0.80-1.12) |
| Human Immunodeficiency Virus Infection | 150 (0.0%) | 48.7% | 1.14 (0.79-1.66) |
|  |  |  |  |
| ELIXHAUSER COMORBIDITY | |  |  |
| Alcohol abuse |  |  |  |
| No | 711,366 (97.5%) | 43.1% | Reference |
| Yes | 18,051 (2.5%) | 42.3% | 1.01 (0.97-1.05) |
| Cardiac Arrhythmia |  |  |  |
| No | 430,820 (59.1%) | 43.3% | Reference |
| Yes | 298,597 (40.9%) | 42.8% | 1.01 (1.00-1.03) |
| Blood Loss Anemia |  |  |  |
| No | 702,439 (96.3%) | 43.1% | Reference |
| Yes | 26,978 (3.7%) | 43.3% | 1.02 (0.99-1.06) |
| Congestive Heart Failure |  |  |  |
| No | 490,360 (67.2%) | 42.8% | Reference |
| Yes | 239,057 (32.8%) | 43.6% | 1.01 (0.99-1.02) |
| Chronic Pulmonary Disease | |  |  |
| No | 471,626 (64.7%) | 42.7% | Reference |
| Yes | 257,791 (35.3%) | 43.8% | 1.01 (1.01-1.03) |
| Coagulopathy |  |  |  |
| No | 663,145 (90.9%) | 43.2% | Reference |
| Yes | 66,272 (9.1%) | 41.8% | 0.98 (0.96-0.99) |
| Deficiency Anemia |  |  |  |
| No | 616,655 (84.5%) | 43.0% | Reference |
| Yes | 112,762 (15.5%) | 43.6% | 1.01 (0.99-1.03) |
| Depression |  |  |  |
| No | 548,028 (75.1%) | 43.1% | Reference |
| Yes | 181,389 (24.9%) | 43.1% | 1.01 (0.99-1.02) |
| Diabetes Complicated |  |  |  |
| No | 535,860 (73.5%) | 43.0% | Reference |
| Yes | 193,557 (26.5%) | 43.3% | 0.99 (0.98-1.01) |
| Diabetes Uncomplicated |  |  |  |
| No | 471,306 (64.6%) | 42.5% | Reference |
| Yes | 258,111 (35.4%) | 44.1% | 1.02 (1.01-1.04) |
| Drug Abuse |  |  |  |
| No | 713,417 (97.8%) | 43.1% | Reference |
| Yes | 16,000 (2.2%) | 42.8% | 1.01 (0.97-1.04) |
| Fluid and Electrolyte Disorders | |  |  |
| No | 464,754 (63.7%) | 43.1% | Reference |
| Yes | 264,663 (36.3%) | 43.0% | 0.99 (0.98-1.01) |
| Acquired Immune Deficiency Syndrome/ Human Immunodeficiency Virus infection | | | |
| No | 728,416 (99.9%) | 43.1% | Reference |
| Yes | 1,001 (0.1%) | 46.6% | 0.99 (0.86-1.15) |
| Hypertension complicated | |  |  |
| No | 493,607 (67.7%) | 43.1% | Reference |
| Yes | 235,810 (32.3%) | 43.2% | 1.01 (0.99-1.03) |
| Hypertension Uncomplicated | |  |  |
| No | 137,786 (18.9%) | 41.8% | Reference |
| Yes | 591,631 (81.1%) | 43.4% | 1.01 (1.00-1.03) |
| Hypothyroidism |  |  |  |
| No | 538,180 (73.8%) | 42.9% | Reference |
| Yes | 191,237 (26.2%) | 43.6% | 1.01 (1.00-1.02) |
| Liver Disease |  |  |  |
| No | 692,354 (94.9%) | 43.1% | Reference |
| Yes | 37,063 (5.1%) | 42.3% | 0.99 (0.97-1.02) |
| Lymphoma |  |  |  |
| No | 713,298 (97.8%) | 43.1% | Reference |
| Yes | 16,119 (2.2%) | 42.3% | 1.02 (0.98-1.06) |
| Metastatic Cancer |  |  |  |
| No | 698,328 (95.7%) | 43.1% | Reference |
| Yes | 31,089 (4.3%) | 41.9% | 1.00 (0.97-1.03) |
| Obesity |  |  |  |
| No | 621,451 (85.2%) | 43.2% | Reference |
| Yes | 107,966 (14.8%) | 42.3% | 0.97 (0.96-0.99) |
| Other Neurological Disorders | |  |  |
| No | 595,708 (81.7%) | 43.2% | Reference |
| Yes | 133,709 (18.3%) | 42.7% | 0.99 (0.98-1.01) |
| Pulmonary Circulation Disorders | |  |  |
| No | 656,412 (89.9%) | 43.2% | Reference |
| Yes | 73,005 (10.1%) | 41.6% | 0.98 (0.97-1.01) |
| Peptic Ulcer Disease excluding bleeding | |  |  |
| No | 715,239 (98.1%) | 43.1% | Reference |
| Yes | 14,178 (1.9%) | 44.7% | 0.99 (0.96-1.03) |
| Peripheral Vascular Disorders | |  |  |
| No | 532,537 (73.0%) | 42.9% | Reference |
| Yes | 196,880(27.0%) | 43.5% | 1.01 (1.00-1.03) |
| Paralysis |  |  |  |
| No | 707,513 (97.0%) | 43.1% | Reference |
| Yes | 21,904 (3.0%) | 43.4% | 0.99 (0.96-1.02) |
| Psychoses |  |  |  |
| No | 708,045 (97.1%) | 42.9% | Reference |
| Yes | 21,372 (2.9%) | 47.8% | 1.03 (0.99-1.07) |
| Renal Failure |  |  |  |
| No | 503,241 (69.0%) | 43.2% | Reference |
| Yes | 226,176 (31.0%) | 42.7% | 0.99 (0.98-1.01) |
| Weight Loss |  |  |  |
| No | 648,514 (88.9%) | 43.0% | Reference |
| Yes | 80,903 (11.1%) | 43.6% | 1.03 (1.01-1.04) |
| Valvular Disease |  |  |  |
| No | 602,203 (82.6%) | 43.3% | Reference |
| Yes | 127,214 (17.4%) | 42.1% | 0.98 (0.97-0.99) |
| Solid Tumor without Metastasis | |  |  |
| No | 625,978 (85.8%) | 43.2% | Reference |
| Yes | 103,439 (14.2%) | 42.3% | 1.01 (0.99-1.02) |
| Rheumatoid Arthritis/collagen | |  |  |
| No | 674,583 (92.5%) | 43.1% | Reference |
| Yes | 54,834 (7.5%) | 42.8% | 1.00 (0.97-1.02) |

**Supplementary eTable 2. The adjusted percent of routine medical admissions receiving all general medical care from one physician.** The analysis is similar to Table 3, except the sample is limited to the 442 major teaching hospitals.

|  |  |  | **Adjusted percent receiving care from one generalist** | | | | | |
| --- | --- | --- | --- | --- | --- | --- | --- | --- |
| **Hospital type** | **N hospital (%)** | **N admission (%)** | **Mean** | **10th** | **25th** | **50th** | **75th** | **90th** |
| **Major Medical School Hospital** | 442 (100%) | 143,993 (100%) | 45.85 | 23.32 | 31.33 | 45.50 | 58.43 | 69.20 |
| **Bed size** |  |  |  |  |  |  |  |  |
| >500 | 184 (41.6%) | 80,952 (56.2%) | 43.48 | 24.60 | 32.21 | 44.56 | 54.31 | 62.92 |
| 201-500 | 195 (44.1%) | 55,712 (38.7%) | 46.36 | 22.92 | 30.39 | 45.43 | 61.34 | 72.29 |
| <=200 | 63 (14.3%) | 7,329 (5.1%) | 53.16 | 21.32 | 31.64 | 54.68 | 71.49 | 86.19 |
| **Location** |  |  |  |  |  |  |  |  |
| Rural | 18 (4.07%) | 2,618 (1.8%) | 61.47 | 33.65 | 43.03 | 63.75 | 81.29 | 89.90 |
| Urban | 424 (95.9%) | 141,375 (98.2%) | 45.39 | 23.26 | 30.81 | 45.11 | 58.02 | 68.29 |
| **Type of provider** | | |  |  |  |  |  |  |
| For profit | 50 (11.3%) | 10,075 (7.0%) | 56.18 | 29.84 | 38.90 | 58.63 | 69.51 | 79.76 |
| Public | 76 (17.2%) | 15,303 (10.6%) | 49.68 | 30.28 | 37.47 | 49.34 | 59.03 | 66.90 |
| Non-profit | 316 (71.5%) | 118,615 (82.4%) | 43.76 | 21.92 | 29.45 | 43.28 | 56.16 | 66.55 |
| **% Medicaid admission** | | | | | | | | |
| Q1 (<=12.8%) | 112 (25.3%) | 46,045 (31.9%) | 42.05 | 20.78 | 27.56 | 42.03 | 54.30 | 65.45 |
| Q2 (>=12.9; <=20.0%) | 123 (27.8%) | 47,849 (33.2%) | 42.33 | 23.58 | 30.18 | 41.22 | 53.99 | 63.79 |
| Q3 (>=12.9; <=30.9%) | 98 (22.2%) | 33,219 (23.1%) | 46.53 | 25.63 | 35.49 | 44.88 | 56.93 | 68.26 |
| Q4 (>=40.0%) | 109 (24.7%) | 16,880 (11.7%) | 54.43 | 25.20 | 38.43 | 58.12 | 67.24 | 79.71 |
